# New York Odontological Society

**Published:** 1892-12

**Authors:** 

**Affiliations:** New York Odontological Society


### Reports of Society Meetings.

### NEW YORK ODONTOLOGICAL SOCIETY.

A REGULAR meeting of the New York Odontological Society was held on Tuesday evening, October 18, 1892, at the New York Academy of Medicine, No. 17 West Forty-third Street, New York City.

The President, Dr. Woodward, occupied the chair.

The minutes of the previous meeting were read and approved.

### INCIDENTS OF OFFICE PRACTICE.

*Dr. Littig.*—I have here a little device which I call the cotton-brush. It is simply made from an ordinary pen-holder and has a wire aluminum screw. I have found it very serviceable in cases where the gums have been very much congested and extremely tender, and in such cases children particularly dislike very much to have their gums brushed with a bristle-brush. These cotton-brushes, which simply consist of twisting a piece of cotton around the screw, clean the teeth very nicely indeed. Of course, the cotton is thrown away after it is used.

I have used these brushes for about eighteen months, and have found they worked very well. Take a piece of cotton and twist the screw, and it winds it on tight, and gives a simple method of carrying an astringent or anything else you want into the mouth. In every case where I treat pyorrhœa alveolaris, I give the patient one of these, and tell him to dip it in listerine, or whatever else I recommend, and rub the gums well.

*Dr. B. C. Nash.*—I would like to relate an incident in my practice, with the idea of drawing out the opinion of the members of this Society. A young miss of thirteen years presented herself, and I found that all of the deciduous teeth had been shed except the right superior second molar. Inasmuch as all of the bicuspids were in position except one, the place of which was occupied by the temporary tooth, I concluded to extract it; but after doing so I failed to find the expected bicuspid under it. The question at once assumed a somewhat serious phase, and I wondered if I had used poor judgment in extracting the deciduous tooth. I did it with some confidence, having had a somewhat similar experience in the case of a young man of twenty-one years, for whom, however, I was able to positively determine the presence of the bicuspid before extracting a temporary molar. My decision was also influenced by another case, that of a lady nearly thirty years of age, who still retains a temporary molar, and the bicuspid which should occupy its position has erupted inside the arch. I have heard the opinion expressed by men of prominence that they would not hesitate to extract under circumstances similar to that I have related. I think it is a question for discussion.

*Dr. Carr.*—Was this molar firm?

*Dr. Nash.*—Yes, tolerably so.

*Dr. Littig.*—I have seen so many cases where the temporary teeth have remained almost during a lifetime, that I do not know whether we would be justified in extracting them, under the circumstances. If the arch were very much crowded we could do so, but for the sake of making the bicuspid erupt, I doubt very much whether it would be the best thing to do.

*Dr. Nash.*—I may say that I have heard expressed the opinions of Dr. Clowes and Dr. Tennison, that they would not hesitate to extract the teeth under those circumstances.

Dr. William Carr then read a paper entitled "Treatment of Fractures of the Maxillæ," illustrating the same with models and appliances.

(For Dr. Carr's paper, see page 849.)

*Dr. Carr.*—If in using the Gibson or the wire splint there is muscular resistance, the reducer invented by Dr. John Meyer, of this city, is indispensable. I have previously objected to using Dr. Gibson's splint on account of the external appliance. But Dr. Gibson assures me that the external portion may be removed with safety to the patient during the day and readjusted at night. In that case the fact of permitting the patient to masticate is of great importance. I have had a set of these splints made, and expect to treat my next case of fracture by this method.

*Dr. Bishop.*—I have had some experience in this line of late years. I have seen a great many cases of fractures, from a blow, from a runaway, from a railroad accident, from a gunshot, from an explosion, and from lightning, and it takes lightning to do the work. I think that it was not only a double and compound fracture, but it was quadruple, and seemed almost too much for a human being to treat.

In all cases that I have seen a simple interdental perforated splint put on the teeth would suffice, and there was no use for the external splint. Dr. Gunning used this splint in 1862, and when it was in the mouth it was received very kindly. The powerful muscles act as an external bandage, and will do all the work necessary. It is very difficult sometimes to make a correct diagnosis. I know of three such cases. A few years ago there was a group of gentlemen standing on the steps of the State House in Boston. One of the gentlemen fell in a fit, and pitched forward on the stone steps. He was carried to his hotel, and was examined by a number of very eminent physicians, who pronounced his injuries fractures about the mouth and jaw; but after a few days, when the bruises and swellings had gone down, it was found there was no fracture whatever. A very prominent man of this city was riding up-town in a Fifth Avenue stage, and as he was getting out, the stage started suddenly and he was thrown forward. He fell on his face, and after being helped home he sent for his surgeon, who pronounced his injuries fractures of the condyles, and put on an external four-tailed bandage, and told him he would have to keep quiet; but his brother-in-law, who was a patient of Dr. Gunning, sent for him. The result of the consultation was that the bandages were removed, and two days afterwards the gentleman went to his business.

Another case was that of a boy who was badly crushed in an elevator accident. A very prominent surgeon in Bellevue Hospital said there was a fracture; the case was postponed for a day or two, and it was found that there was no fracture whatever.

A surgeon in treating a bone of the leg or the arm can get around it; but in the lower maxilla he gets on the wrong side of it; we, however, can get on the right side of that bone, and we make the muscles and the interdental splint do the work, and, as Dr. Carr says, a man may not even be detained from his business, but can go right along, and the bone unites and makes a perfect union.

*Dr. Carr.*—Don't you think that the surgeon gave the proper treatment in putting on the bandages, in the case that you mentioned?

*Dr. Bishop.*—I think not.

*Dr. Carr.*—Was not the object of the bandage to keep the muscles at rest? Was not that good treatment?

*Dr. Bishop.*—No; I don't think so. Sometimes you displace a fracture very much by external bandages.

*Dr. Littig.*—In regard to fractures of the superior maxilla, they are a matter of little moment, so far as I can see, and it takes very little to keep them in position. The first case of that kind that I ever had was one of an old lady who was wearing an artificial set of teeth, and by falling she struck the side of her face against a silver tea-pot which she was holding in her hand. When I saw her, the alveolar ridge on the right side was in about the centre of the mouth. She was bleeding quite profusely, and I sent for some styptic cotton and applied it, which arrested the hemorrhage. She had a considerable cut on the cheek, which I drew together with adhesive plaster. Tincture of calendula was recommended as a wash for the mouth, and I told her to wear her plate, which she did. Although she was almost eighty years old at the time, there was perfect adhesion of the bone, and she was able to wear her teeth just the same as ever. As Dr. Carr said in one of his cases, it is very peculiar that in the fracture the pulps of the teeth were preserved alive. Usually a blow of such great force would destroy them. In that case the blow must have been so much above the points of the teeth, that it carried the alveolus and the jaw-bone right over, and did not destroy the pulps.

*Dr. Davenport.*—Is plaster ever used in taking impressions?

*Dr. Carr.*—Yes, but I never use it in these cases. I find that I can take an accurate impression with the compound.

*Dr. Bishop.*—If I may answer that question, I would say that I should take it, by all means, in plaster. The compound or wax makes too much pressure. If you have the right cup and oil it, and let the plaster stay until it gets thoroughly hard, you can then remove your cup, and the plaster is still in place. Now use a lance-knife and cut through the plaster to the tooth, then down the side of a canine or bicuspid, then with a chisel it is easily broken and removed in sections; when placed back into the cup you have a perfect impression. The trouble is, it fits too nicely. In vulcanizing the splint, use tin-foil over the cast, thus producing greater freedom in getting it into place.

*Dr. Hill.*—Is your only objection to the compound the pressure that it causes?

*Dr. Bishop.*—Yes; Dr. Gunning had in his day seven different sizes of jaw-cups made. They are deeper than this splint that Dr.

Carr has shown us, and larger. As already stated, you can fit those to your jaw, lower or upper, very nicely; then by oiling the inside, and using the plaster very soft, you will not cause any pain by pressure. You will find these sections of the impression go right back into the cup.

Dr. Gunning's idea was that you might use gutta-percha in the same cup, after getting your cast, and thus make a splint of it; but I prefer to use vulcanite splints, perforated so that when they are in place the tongue and the muscles of the cheek are constantly passing fluids through, and with a brush they can be kept very clean.

*Dr. Carr.*—Does not the gutta-percha splint become very foul in three or four days?

*Dr. Bishop.*—Yes; that is the objection, but with the vulcanite splint it is very sweet. These cups are very useful in taking impressions of the lower jaw.

*Dr. Perry.*—I do not know that I ought to bring this subject before the Society, but I wish to say a few words. I received a letter from Dr. Barrett, in which he asks permission of the Society to have published an address which he made at the dinner given him on the presentation of the osteological collection now in our rooms. He would like to have the official sanction of the Society to have the paper published in the *Dental Review.*

On motion, privilege was granted to Dr. Barrett to have the address published.

After a vote of thanks had been tendered to Dr. Carr for his interesting paper, the meeting adjourned.

John I. Hart, D.D.S.,
*Editor New York Odontological Society.*

